# A Bayesian Network to Predict the Risk of Post Influenza Vaccination Guillain-Barré Syndrome: Development and Validation Study

**DOI:** 10.2196/25658

**Published:** 2022-03-25

**Authors:** Yun Huang, Chongliang Luo, Ying Jiang, Jingcheng Du, Cui Tao, Yong Chen, Yuantao Hao

**Affiliations:** 1 Department of Medical Statistics Sun Yat-Sen University Guangzhou China; 2 Guangdong Provincial Center for Disease Control and Prevention Guangzhou China; 3 Department of Biostatistics, Epidemiology and Informatics University of Pennsylvania Philadelphia, PA United States; 4 Division of Public Health Sciences Washington University School of Medicine in St. Louis St. Louis, MO United States; 5 Department of Neurology and Multiple Sclerosis Research Center, The Third Affiliated Hospital Sun Yat-Sen University Guangzhou China; 6 School of Biomedical Informatics The University of Texas Health Science Center at Houston Houston, TX United States; 7 Peking University Center for Public Health and Epidemic Preparedness & Response Beijing China

**Keywords:** adverse events, Bayesian network, Guillain-Barré syndrome, risk prediction, trivalent influenza vaccine

## Abstract

**Background:**

Identifying the key factors of Guillain-Barré syndrome (GBS) and predicting its occurrence are vital for improving the prognosis of patients with GBS. However, there are scarcely any publications on a forewarning model of GBS. A Bayesian network (BN) model, which is known to be an accurate, interpretable, and interaction-sensitive graph model in many similar domains, is worth trying in GBS risk prediction.

**Objective:**

The aim of this study is to determine the most significant factors of GBS and further develop and validate a BN model for predicting GBS risk.

**Methods:**

Large-scale influenza vaccine postmarketing surveillance data, including 79,165 US (obtained from the Vaccine Adverse Event Reporting System between 1990 and 2017) and 12,495 European (obtained from the EudraVigilance system between 2003 and 2016) adverse events (AEs) reports, were extracted for model development and validation. GBS, age, gender, and the top 50 prevalent AEs were included for initial BN construction using the R package *bnlearn*.

**Results:**

Age, gender, and 10 AEs were identified as the most significant factors of GBS. The posttest probability of GBS suggested that male vaccinees aged 50-64 years and without erythema should be on the alert or be warned by clinicians about an increased risk of GBS, especially when they also experience symptoms of asthenia, hypesthesia, muscular weakness, or paresthesia. The established BN model achieved an area under the receiver operating characteristic curve of 0.866 (95% CI 0.865-0.867), sensitivity of 0.752 (95% CI 0.749-0.756), specificity of 0.882 (95% CI 0.879-0.885), and accuracy of 0.882 (95% CI 0.879-0.884) for predicting GBS risk during the internal validation and obtained values of 0.829, 0.673, 0.854, and 0.843 for area under the receiver operating characteristic curve, sensitivity, specificity, and accuracy, respectively, during the external validation.

**Conclusions:**

The findings of this study illustrated that a BN model can effectively identify the most significant factors of GBS, improve understanding of the complex interactions among different postvaccination symptoms through its graphical representation, and accurately predict the risk of GBS. The established BN model could further assist clinical decision-making by providing an estimated risk of GBS for a specific vaccinee or be developed into an open-access platform for vaccinees’ self-monitoring.

## Introduction

### Background

Influenza vaccine is currently the most effective intervention to prevent millions of influenza-related visits to the physician each year [1]. Although the benefits of getting vaccinated far outweigh its risks, influenza vaccine is occasionally associated with adverse events (AEs), and as with most of medicine, there is a very rare chance of an influenza vaccine causing a severe reaction [1]. Guillain-Barré syndrome (GBS) is the most common and most severe acute paralytic neuropathy [2] that develops in susceptible individuals after infection and, in rare cases, after immunization (including influenza vaccination) [3]. The estimated incidence of GBS among the general population ranges from 0.8 to 1.9 cases per 100,000 person-years [4]. Although some epidemiological studies suggested that there may be a very small increased risk of GBS after influenza vaccination [5,6], causality remains controversial [3,7,8] and is out of the scope of this study. The identification of GBS is largely based on clinical patterns [2], and meticulous monitoring, supportive care, and the early start of specific treatment are necessary for patients with GBS [9]. Therefore, determining the key factors of GBS and predicting its occurrence are vital for improving the prognosis of these patients.

The Vaccine Adverse Event Reporting System (VAERS), comanaged by the Centers for Disease Control and Prevention and the US Food and Drug Administration, is a nationwide passive surveillance program to detect possible safety problems for US-licensed vaccines [10]. VAERS accepts reports of postvaccination AEs from 1990 to the present and collects information such as vaccinees’ age, gender, the experienced AEs, and the recovery status. A primary objective of VAERS is to monitor fluctuations in known AEs that might indicate a potential safety problem with a vaccine [10]. GBS is one such concern and is the targeted AE of this study. Previous studies of GBS onset based on VAERS data reported that GBS generally occurs 2 weeks after influenza vaccination, which is later than that of most other influenza vaccine–related AEs [11,12]. Besides, some clinical features (eg, muscular weakness, pain, and autonomic dysfunction) that can be used to identify GBS [13] are also recorded as separate AEs in VAERS. Thus, performing a deep data mining of VAERS and identifying the most informative GBS-related AEs is significant and valuable work. The identified AEs first help in forming a future study hypothesis for etiological research of GBS and then can further be used to develop risk-prediction models that enable early warning.

Existing efforts focus on the measurement and prediction of clinical course and outcome of GBS, and good prognostic models have been developed [14-17]. However, as far as we know, there is no publication on a forewarning model of GBS, except for our previous work [18], which constructed a multivariate logistic regression model using GBS-related AEs in VAERS to predict risk of GBS. Nevertheless, conventional linear models (eg, multiple linear regression model and logistic regression model) may be biased in dealing with collinearity and complex interactions when analyzing multiple predictors. In addition, it is difficult to succinctly present or explain the subtle patterns behind a particular prediction with general machine learning methods (eg, artificial neural network and support vector machine).

### Bayesian Network Model

A Bayesian network (BN) is an emerging type of probabilistic graph model for predicting risk of outcomes of interest [19]. As a well-established type of probabilistic classifier, a BN model has the advantages of identifying interactions among variables that are often neglected by conventional statistical models and outputting an intuitive conditional probability table (CPT) for decision-making. In addition, the Markov blanket (MB) theory gives BN models the capacity for identifying the most significant factors contributing to the outcome. BN models have been applied for predicting risk of AEs in, among many others, radiotherapy [20] and hemodialysis [21] and have previously been shown to perform well at predicting the risk of other diseases using electronic health records [22-24]. However, whether a BN model can identify the most significant factors of GBS and integrate them to predict GBS remains to be determined.

Because of the rarity of many postvaccination AEs, especially for GBS, many longitudinal studies or cohort studies are underpowered in identifying risk factors for early detection. The large amount of data accumulated since 1990 in VAERS provides an opportunity for such studies: among influenza vaccine–related VAERS reports, trivalent influenza vaccine (FLU3)-related VAERS reports compose a major portion. The purpose of our investigation is to identify the most significant factors of GBS using FLU3-related VAERS reports, generate a novel risk prediction model, and estimate the probability of GBS occurrence. This study was reported following the Transparent Reporting of a Multivariable Prediction Model for Individual Prognosis or Diagnosis statement [25] (Multimedia Appendix 1). For a specific vaccinee who has certain AEs, the estimated risk from the risk prediction models could help to measure the risk of GBS and allow for timely diagnosis and treatment. We believe our work is complementary to other investigations and could ultimately lead to useful insights for clinical decision-making.

## Methods

### Data Processing

The VAERS database had received more than 400,000 vaccine-associated AE reports by the end of 2018. Each report had been manually annotated at the preferred-term level in the Medical Dictionary for Regulatory Activities by domain experts. We extracted all the FLU3-related VAERS reports between 1990 and 2017. The reports were excluded if they met either of the following criteria: (1) missing age values or age <0.5 years and (2) unknown gender status. We finally included 79,165 completed reports and 2978 unique AE symptoms, including GBS.

### Ethics Approval and Consent to Participate

Ethics approval and consent to participate are not applicable to this study because the VAERS database we used is publicly available [26]. The EudraVigilance vaccine AE data were requested from the European Medicines Agency.

### Learning a BN

A BN *B* can be defined as a pair [27]:

B(G,Θ), G = (V,E)

Here, *G* = (*V,E*) is a directed acyclic graph that encodes the structure of the BN, in which each node *X_i_* in *V* corresponds to a domain variable (discrete or continuous) and *E* consists of a set of directed arcs (or edges) that connect pairs of nodes. As in a genealogical chart, a parent node points to a child node with a directed arc, and an arc between 2 variables indicates a relationship of direct dependence. Furthermore, the Markov property states that any node *X* is conditionally independent of any other nodes, given its MB, and the MB of a node includes its parents, its children, and the children’s other parents (spouses). *Θ* is a set of parameters that quantify the graph edges by specifying the conditional probability distributions; in the discrete case, they are denoted as CPTs. The joint probability distribution *P* factorized as a product of multiple conditional probability distributions also denotes the dependency or independency structure of the directed acyclic graph:



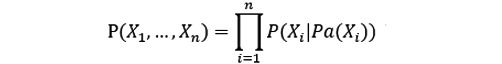



Here, *Pa*(*X_i_*) represents the parent nodes of *X_i_*.

Accordingly, the process of learning a BN can be separated into two steps: BN structure learning and BN parameter learning. Many state-of-the-art BN structure learning algorithms have been proposed to determine the topology of a BN from data, and maximum likelihood estimation (MLE) and Bayesian parameter estimation are two popular methods for parameter learning. In addition, prior knowledge of the structure or parameters can also be integrated into the BN learning process.

### Statistical Analysis

Age was discretized into four groups: 0.5-17, 18-49, 50-64, and ≥65 years. All AEs were binary variables, with status *true* or *false* indicating whether the AE occurred or did not occur, respectively. We sorted all the AEs by their prevalence in the US data and selected the top 50 for further analysis (we also performed the analysis with the top 100 AEs to compare different networks). The prevalence of the top 50 AEs was compared between the GBS group and the non-GBS group using the Pearson chi-square test. To avoid inflating type I error caused by multiple comparisons, a 2-sided *P* value <.001 (Bonferroni correction) was used to indicate a statistically significant difference.

**Figure 1:**
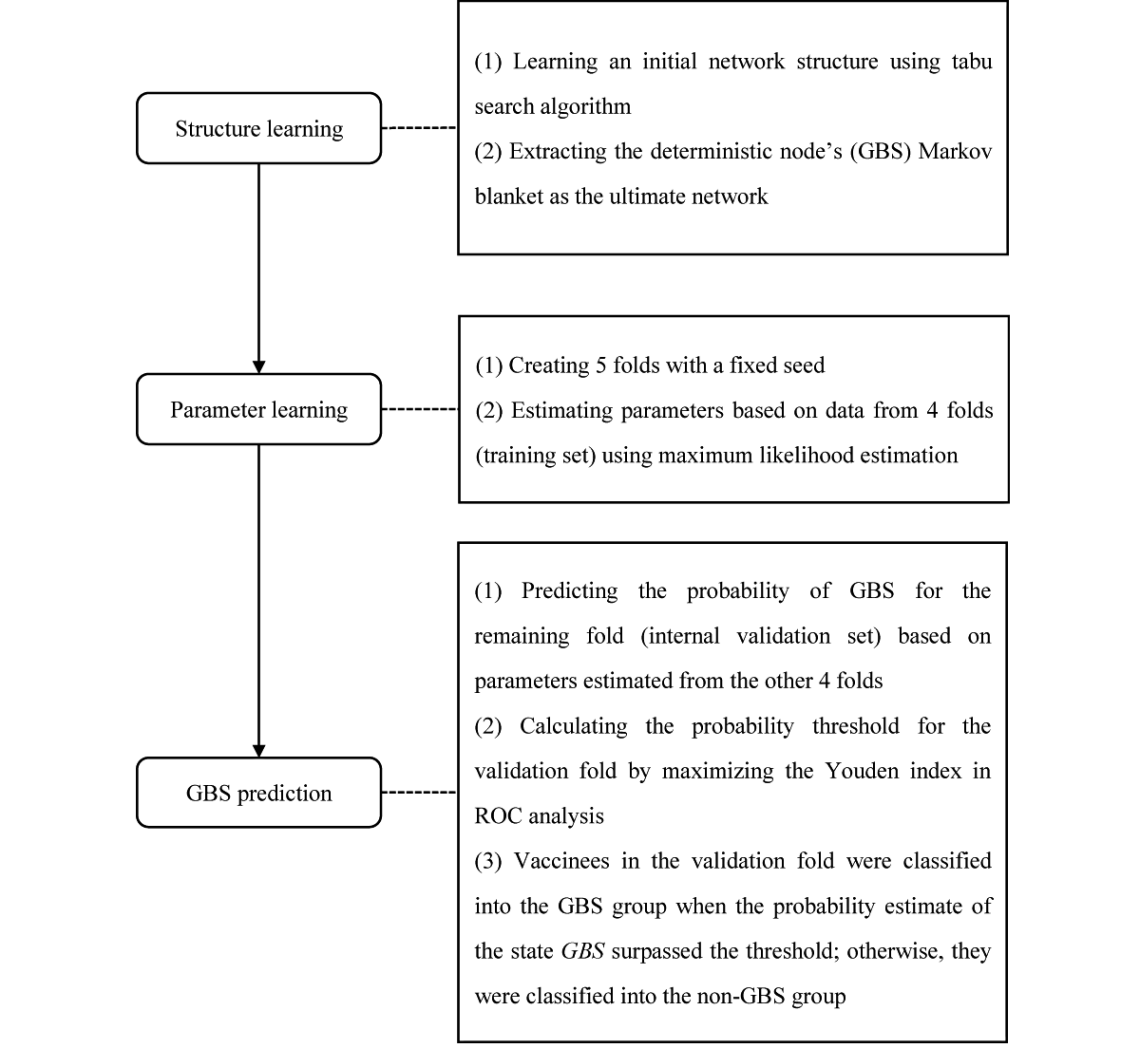
Flow diagram of Bayesian network learning. GBS: Guillain-Barré syndrome; ROC: receiver operating characteristic curve.

GBS was set as the deterministic node, and all 53 variables (GBS, age, gender, and the top 50 AEs) were included in construction of the initial network. The flow diagram of BN learning is shown in Figure 1. Tabu search is a higher-level heuristic procedure that maintains the advantage of score-based structure learning algorithms and escapes the trap of local optimality [28]. For the first step, we obtained an initial network structure using the tabu search algorithm, with setting as a blacklist of arcs (no other variable can point to age or gender) and a whitelist of arcs (both age and gender point to GBS) based on prior knowledge [2]. Generally, we should consult domain experts to adjust those illogical arcs in the initial network to obtain a more reasonable structure. However, many variables were included in the initial network; therefore, we chose to extract the MB of GBS as the ultimate network from the perspective of model complexity and the Markov property. The strength of the conditional-dependence relationships among nodes was measured by Bayesian information criterion score gain or loss that would be caused by each arc’s removal. For the second step, we performed 5-fold cross-validation 100 times, learned parameters using MLE based on 4 folds (ie, training folds), and obtained CPTs quantifying the probability of each state of a node based on all possible combinations of its parent nodes’ values. In a discrete BN such as the one used in this study, parameters learned by MLE are approximately equal to the frequency of specific value of a node in the training data when fixing its parent nodes’ values. Finally, we predicted the probability of GBS for the remaining fold (ie, validation fold) based on parameters estimated from training folds and calculated the probability threshold for the validation fold by maximizing the Youden index in the receiver operating characteristic curve analysis. Vaccinees in the validation fold were classified into the GBS group when the probability estimates of the state *GBS* surpassed the threshold; otherwise, they were classified into the non-GBS group.

Area under the receiver operating characteristic curve (AUC), sensitivity, specificity, and predictive accuracy were used to assess the performance of the established BN. Here, sensitivity implies the ability of a model to identify a patient as a positive result, specificity implies the ability of a model to identify a nonpatient as a negative result, AUC is a comprehensive index that integrates a model’s sensitivity and specificity, and accuracy implies the ability of a model to correctly identify both patient and nonpatient. The results of internal validation folds were averaged to obtain the ultimate indices, and their 95% CIs were calculated using the approximate normal distribution method. R (version 4.0.0; The R Foundation for Statistical Computing) packages, including *bnlearn*, *pROC*, *gmodels*, and *caret*, were used for the statistical analyses.

### External Validation

The performance of the BN established from the US data was validated using the European EudraVigilance data. The European data were obtained from the European Medicines Agency in 2016 and included AE reports following influenza vaccines from 2003 to 2016. We filtered out records with missing age values or unknown gender status, as well as those reported outside the European Union area, and finally a total of 12,495 completed records were extracted. It is worth mentioning that the European data covered not only FLU3 but also other influenza vaccines such as quadrivalent influenza vaccine and monovalent influenza vaccine (H1N1 influenza vaccine) because of data access limitation and the formulation of FLU3 in Europe is different from that in the United States.

The same set of variables as in the US data (GBS, age, gender, and the top 50 AEs) were extracted from the European data, and age was also discretized into 4 groups as stated in the *Statistical Analysis* section. During the external validation procedure in the European data, we applied the BN structure and its parameters learned from all the US data to predict the probability of GBS in European vaccinees and categorized them into two classes (GBS and non-GBS) as we did in the internal validation folds. The same performance metrics were applied.

## Results

### Descriptive Analysis

On the basis of the VAERS and EudraVigilance data, the cumulative probability of GBS was 1.26% within 28 years following the US FLU3 vaccine and 1.71% within 14 years following all the European flu vaccines (Table 1). For the US population, the median age of the GBS group was higher than that of the non-GBS group (median 57, IQR 42-68 years vs median 50, IQR 29-66 years), and this trend was similar and more obvious in the European population (median 60, IQR 49.25-72.00 years vs median 46, IQR 22.00-64.00 years). For the GBS reporters in both the United States and Europe, the percentage of 4 age groups increased gradually and this disease was slightly more frequent in men than in women, both of which were consistent with previous studies [2].

Among the top 50 AEs, 33 (66%) presented significant association with GBS in the US data, whereas only 9 (18%) presented significant association with GBS in the European data (Table 2). Of the AEs significantly associated with GBS, only 15% (5/33; asthenia, fatigue, paresthesia, hypesthesia, and muscular weakness) showed a positive association in the US data, whereas 100% (9/9) of the significant AEs (pain, pain in extremity, asthenia, fatigue, paresthesia, hypesthesia, tremor, musculoskeletal pain, and muscular weakness) showed positive association in the European data, with these 9 AEs including the aforementioned 5 AEs. As for the total prevalence of the top 50 AEs, only 12 (24%) had no significant difference between the United States and Europe.

**Table 1:** Demographic characteristics of 79,165 US (Vaccine Adverse Event Reporting System, trivalent influenza vaccine [FLU3], 1990-2017) reports and 12,495 European reports (EudraVigilance, all flu vaccines, 2003-2016).

Characteristics			GBS^a^	Non-GBS	Total
**US FLU3 vaccine reports, 1990-2017 (N=79,165)**					
	Number of reports, n (%)		996 (1.26)	78,169 (98.74)	79,165 (100)
	**Age (years), median (IQR)**		57 (42-68)	50 (29-66)	50 (29-66)
		0.5-17	57 (5.72)	13,199 (16.89)	13,256 (16.74)
		18-49	276 (27.71)	25,146 (32.17)	25,422 (32.11)
		50-64	326 (32.73)	17,147 (21.94)	17,473 (22.07)
		≥65	337 (33.84)	22,677 (29.01)	23,014 (29.07)
	Men, n (%)		505 (50.70)	23,327 (29.84)	23,832 (30.10)
**European flu vaccine reports, 2003-2016 (N=12,495)**					
	Number of reports, n (%)		214 (1.71)	12,281 (98.29)	12,495 (100)
	**Age (years), median (IQR)**		60 (49.25-72)	46 (22-64)	46 (22-65)
		0.5-17	6 (2.80)	2691 (21.91)	2697 (21.58)
		18-49	48 (22.43)	3987 (32.46)	4035 (32.29)
		50-64	74 (34.58)	2548 (20.75)	2622 (20.98)
		≥65	86 (40.19)	3055 (24.88)	3141 (25.14)
	Men, n (%)		125 (58.41)	4949 (40.30)	5074 (40.61)

^a^GBS: Guillain-Barré syndrome.

**Table 2:** Top 50 prevalent adverse events screened from the US data, and comparisons between the US and European data.

Adverse events	US FLU3^a^ reports, 1990-2017					European flu vaccine reports, 2003-2016					US vs Europe, *P* value, total
	Total (‰)	GBS^b^ (‰)	Non-GBS (‰)	*P* value	Total (‰)		GBS (‰)	Non-GBS (‰)	*P* value		
Pyrexia	138.85	58.23	139.88	<.001	140.86		79.44	141.93	.01	.55	
Injection-site erythema	126.36	2.01	127.94	<.001	15.53		0	15.80	.06	<.001	
Pain	125.40	113.45	125.55	.25	29.77		112.15	28.34	<.001	<.001	
Injection-site pain	119.16	7.03	120.58	<.001	30.97		4.67	31.43	.03	<.001	
Erythema	89.69	3.01	90.79	<.001	23.37		4.67	23.70	.07	<.001	
Pain in extremity	86.79	73.29	86.97	.13	46.74		177.57	44.46	<.001	<.001	
Injection-site swelling	86.07	3.01	87.13	<.001	12.48		0	12.70	.10	<.001	
Headache	77.52	61.24	77.73	.05	103.16		88.79	103.41	.49	<.001	
Pruritus	71.33	2.01	72.22	<.001	23.53		18.69	23.61	.64	<.001	
Chills	68.46	22.09	69.06	<.001	37.21		32.71	37.29	.73	<.001	
Dizziness	63.45	32.13	63.85	<.001	60.02		65.42	59.93	.74	.14	
Nausea	62.70	25.10	63.18	<.001	55.14		23.36	55.70	.04	.001	
Urticaria	60.75	2.01	61.49	<.001	27.85		0	28.34	.01	<.001	
Rash	59.50	10.04	60.13	<.001	26.57		9.35	26.87	.11	<.001	
Injection-site warmth	55.91	< 0.01	56.62	<.001	4.08		0	4.15	.34	<.001	
Dyspnea	50.67	57.23	50.58	.34	47.94		70.09	47.55	.13	.19	
Myalgia	49.52	49.20	49.52	.96	66.43		56.07	66.61	.54	<.001	
Vomiting	45.60	26.10	45.85	.003	42.26		37.38	42.34	.72	.09	
Asthenia	44.78	296.18	41.58	<.001	39.62		121.50	38.19	<.001	.01	
Fatigue	37.96	59.24	37.69	<.001	72.91		135.51	71.82	<.001	<.001	
Paresthesia	37.57	332.33	33.81	<.001	43.06		345.79	37.78	<.001	.003	
Cough	37.16	32.13	37.23	.40	38.02		23.36	38.27	.26	.64	
Edema, peripheral	35.20	9.04	35.54	<.001	5.52		4.67	5.54	.87	<.001	
Malaise	32.22	27.11	32.29	.36	55.62		56.07	55.61	.98	<.001	
Skin, warm	31.34	1.00	31.73	<.001	1.92		0	1.95	.52	<.001	
Hypesthesia	31.31	289.16	28.03	<.001	26.65		285.05	22.15	<.001	.005	
Swelling	30.76	3.01	31.11	<.001	5.28		0	5.37	.28	<.001	
Arthralgia	28.59	19.08	28.71	.07	39.06		37.38	39.08	.90	<.001	
Injection-site edema	28.09	1.00	28.44	<.001	2.40		0	2.44	.47	<.001	
Injection-site hypersensitivity	26.38	2.01	26.69	<.001	0.24		0	0.24	.82	<.001	
Diarrhea	24.30	24.10	24.31	.97	27.29		42.06	27.03	.18	.05	
Hyperhidrosis	23.08	7.03	23.28	<.001	18.65		9.35	18.81	.31	.002	
Tremor	21.82	14.06	21.91	.09	9.68		32.71	9.28	<.001	<.001	
Injection-site pruritus	21.26	< 0.01	21.53	<.001	3.60		0	3.66	.38	<.001	
Injected-limb mobility decreased	20.78	2.01	21.02	<.001	2.96		0	3.01	.42	<.001	
Feeling hot	19.88	< 0.01	20.14	<.001	10.40		9.35	10.42	.88	<.001	
Injection-site reaction	19.39	1.00	19.62	<.001	8.48		0	8.63	.17	<.001	
Musculoskeletal pain	18.91	12.05	19.00	.11	11.20		46.73	10.59	<.001	<.001	
Injection-site induration	18.86	< 0.01	19.10	<.001	4.16		0	4.23	.34	<.001	
Cellulitis	18.63	1.00	18.86	<.001	2.16		0	2.20	.49	<.001	
Muscular weakness	17.75	230.92	15.03	<.001	25.13		266.36	20.93	<.001	<.001	
Vasodilatation	17.61	2.01	17.81	<.001	0.32		0	0.33	.79	<.001	
Neck pain	17.51	16.06	17.53	.73	7.20		4.67	7.25	.66	<.001	
Mobility decreased	16.32	24.10	16.22	.05	3.92		14.02	3.75	.02	<.001	
Immediate postinjection reaction	16.28	3.01	16.45	<.001	0		0	0	—^c^	—	
Chest pain	16.21	18.07	16.18	.64	13.93		14.02	13.92	.99	.06	
Rash, erythematous	15.61	2.01	15.79	<.001	4.88		0	4.97	.30	<.001	
Injection-site rash	15.45	1.00	15.63	<.001	1.36		0	1.38	.59	<.001	
Syncope	15.42	8.03	15.52	.06	29.21		4.67	29.64	.03	<.001	
Tenderness	15.40	3.01	15.56	.001	1.20		4.67	1.14	.14	<.001	

^a^FLU3: trivalent influenza vaccine.

^b^GBS: Guillain-Barré syndrome.

^c^Not available.

### The Established BN

**Figure 2:**
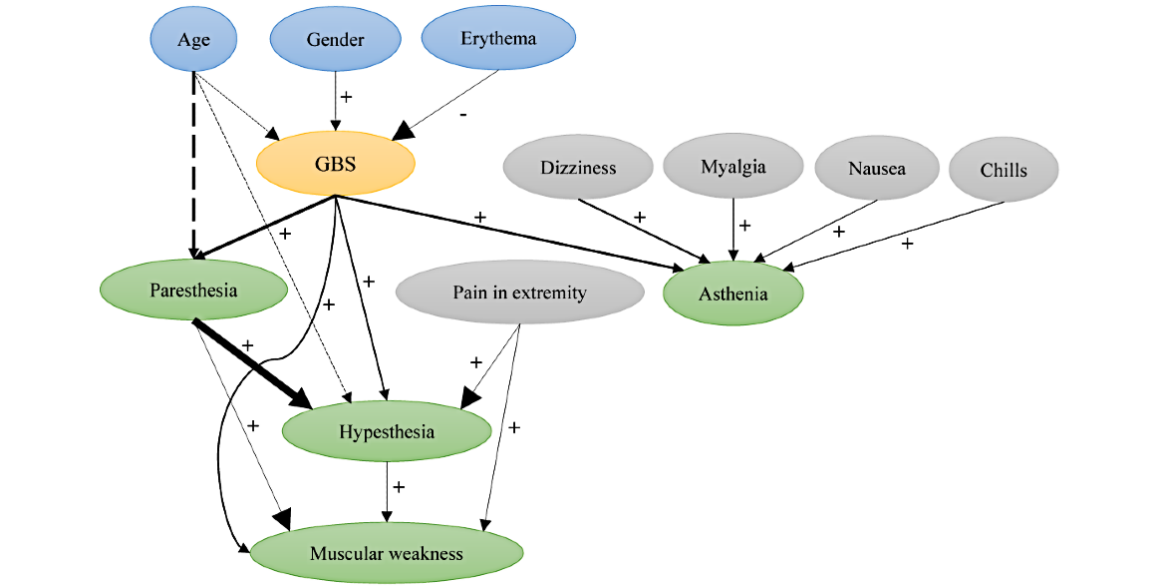
Structure of the established Bayesian network. Labeled ovals represent nodes; arrows (arcs) represent conditional-dependence relationships. The oval in yellow represents the deterministic node, ovals in blue represent the deterministic node’s parent nodes, ovals in green represent the child nodes, and ovals in gray represent the spouse nodes. Arc thickness is proportional to the strength of the conditional-dependence relationship. Minus (–) or plus (+) sign indicates either negative or positive association, respectively, between the nodes; arcs in dashed lines indicate a U-shaped association between 2 nodes. GBS: Guillain-Barré syndrome.

The MB of the GBS, that is, the ultimate network structure (Figure 2), contained three parent nodes (age, gender, and erythema), four child nodes (asthenia, hypesthesia, muscular weakness, and paresthesia), and five spouse nodes (chills, dizziness, myalgia, nausea, and pain in extremity), and they were the most significant factors of GBS. Among these, age also played a spouse node role when it coacted with GBS in influencing the occurrence of paresthesia and hypesthesia. Besides, paresthesia was also a spouse node that interacted with GBS to influence hypesthesia and muscular weakness, and hypesthesia also acted as a spouse of GBS in influencing muscular weakness. As an arc pointing from a parent node to a child node indicates a chronological order, we may learn from the MB that age, gender, and erythema acted on the occurrence of GBS; subsequently, GBS interacted with the spouse nodes and further evolved into symptoms of asthenia, hypesthesia, muscular weakness, and paresthesia. The remaining AEs (40/50, 80%) were conditionally independent of GBS through the nodes in the MB and were pruned to retain a more compact network.

The arc thickness in Figure 2 is proportional to the strength of the conditional-dependence relationship; it serves to show that the conditional-dependence relationship between paresthesia and hypesthesia was the strongest, followed by that between GBS and paresthesia, between age and paresthesia, and between GBS and asthenia (detailed strength values can be found in Table S1 in Multimedia Appendix 2). Furthermore, we found that most of the parent nodes had a positive correlation with their child nodes in our network, except for the negative correlation between erythema and GBS, and the risk of GBS, paresthesia, and hypesthesia first increased and then decreased with increasing age.

### Posttest Probability of the Deterministic Node

**Figure 3:**
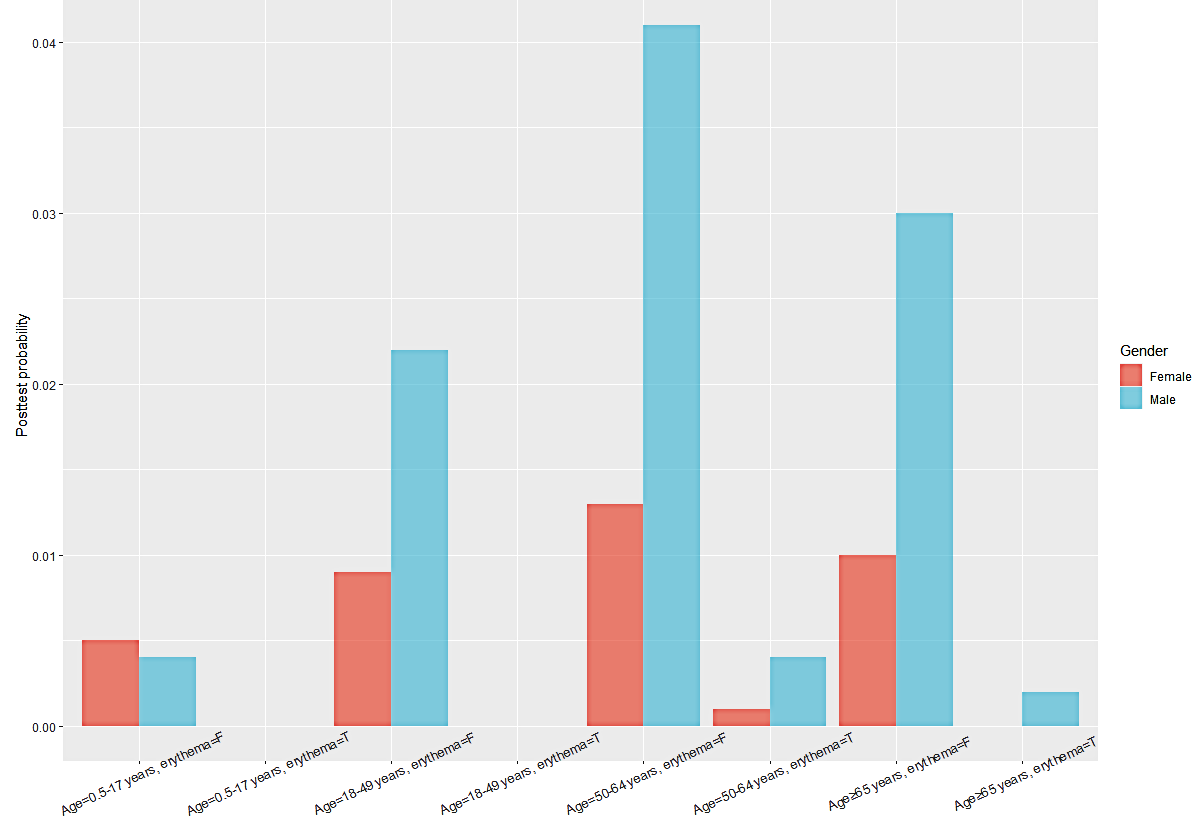
Posttest probability of the deterministic node based on its parent nodes’ combinations. F: false; T: true.

The posttest probability of GBS based on the status of its 3 parent nodes is shown in Figure 3. It suggested that male vaccinees aged 50-64 years and without erythema had the highest probability of acquiring GBS, followed by vaccinees aged ≥65 years or those aged 18-49 years, with the other 2 features remaining unchanged. Female vaccinees aged 50-64 years and without erythema also tended to experience GBS, but male vaccinees in the same situation had almost triple the risk. In contrast, vaccinees with different other combinations of the aforementioned 3 parent nodes showed a reduced probability of acquiring GBS. Vaccinees who experienced the AE of erythema were estimated to have almost no chance of acquiring GBS.

### Performance of the BN

The constructed BN model performed desirably at predicting GBS, with an AUC of 0.866 (95% CI 0.865-0.867), sensitivity of 0.752 (95% CI 0.749-0.756), specificity of 0.882 (95% CI 0.879-0.885), and accuracy of 0.882 (95% CI 0.879-0.884) at a probability threshold of 0.014 (95% CI 0.0136-0.0143) for the internal validation. The best performance of the BN during cross-validation reached an AUC of 0.906. As for the external validation in the European data, the established BN obtained values of 0.829, 0.673, 0.854, and 0.843 for AUC, sensitivity, specificity, and accuracy, respectively.

### BN Development and Validation With the Top 100 AEs

**Figure 4:**
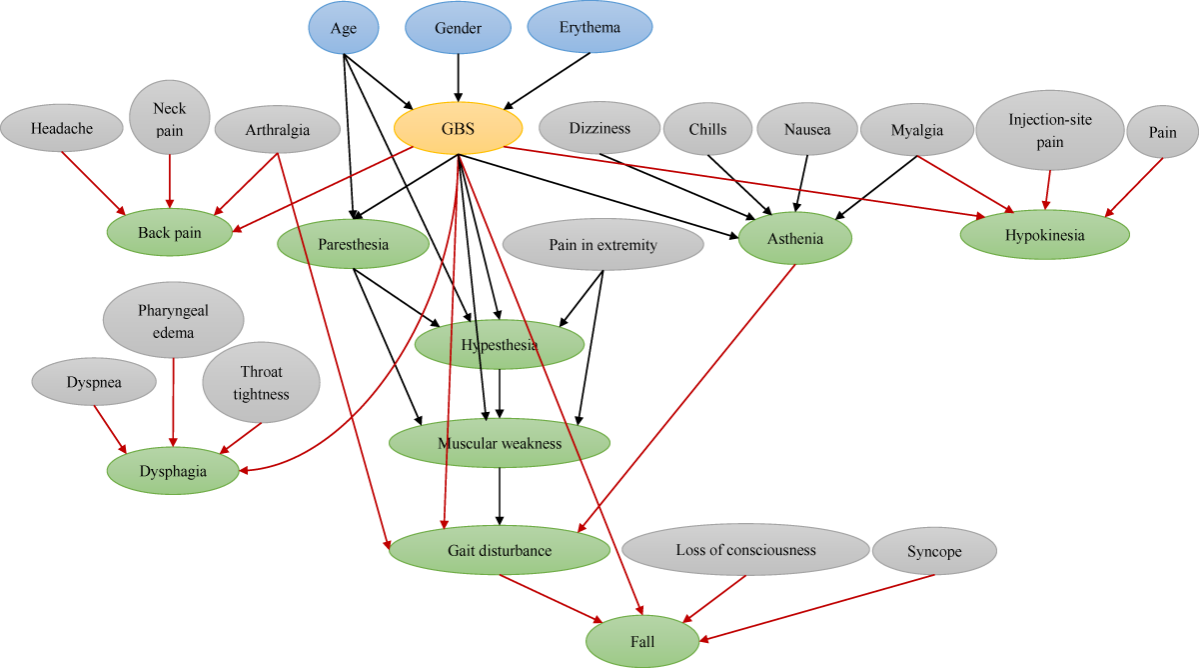
The Bayesian network structure established from the top 100 adverse events. Labeled ovals represent nodes; arrows (arcs) represent conditional-dependence relationships. The oval in yellow represents the deterministic node, ovals in blue represent the deterministic node’s parent nodes, ovals in green represent the child nodes, and ovals in gray represent the spouse nodes. Arcs in red indicate the added arcs in the new structure compared with the Bayesian network structure learned from the top 50 adverse events. GBS: Guillain-Barré syndrome.

The BN structure learned from the top 100 AEs (Figure 4) contained the structure learned from the top 50 AEs. Compared with the BN structure learned from the top 50 AEs, there were 5 more child nodes (back pain, dysphagia, fall, gait disturbance, and hypokinesia) and 10 more spouse nodes (headache, neck pain, arthralgia, injection-site pain, pain, dyspnea, pharyngeal edema, throat tightness, loss of consciousness, and syncope) in the new structure.

The new BN model had a slightly improved performance compared with the previous one, obtaining an AUC of 0.883 (95% CI 0.881-0.884), sensitivity of 0.787 (95% CI 0.784-0.790), specificity of 0.891 (95% CI 0.889-0.893), and accuracy of 0.890 (95% CI 0.888-0.892) at a probability threshold of 0.012 (95% CI 0.0115-0.0122) for the internal validation and achieving values of 0.832, 0.664, 0.915, and 0.911 for AUC, sensitivity, specificity, and accuracy, respectively, in the external validation.

## Discussion

### Principal Findings

BN models are highly attractive because of their ability to describe complex probabilistic interactions among variables and to determine a unique joint probability distribution over multiple variables for probabilistic inference. In this study, we identified the 10 most informative GBS-related AEs from the MB of GBS; constructed the joint probability distribution based on age, gender, and these 10 AEs to predict the likelihood of GBS; and achieved a desirable performance.

In accordance with previous studies [2,13], the established BN structure also suggested sensory signs of asthenia, hypesthesia, muscular weakness, and paresthesia as clinical features of GBS. Besides, it recommended that age, gender, and erythema should also be taken into account for identifying GBS in clinical practice. Although many epidemiological studies have reported increased age and male gender as risk factors for GBS [29-32], none took these two demographic characteristics as a basis for identification of GBS occurrence. Our efforts may promote the advancement of precision medicine in GBS identification. Furthermore, the symptom of erythema, which is an observable and not easily overlooked body sign, provides more explicit information than sensory signs for vaccinees or clinicians to evaluate GBS risk. Moreover, additional symptoms of chills, dizziness, myalgia, nausea, and pain in extremity should also raise doubt about an increased risk of GBS; these symptoms have been presented in many case reports [33-35] but have not been used for GBS identification. In addition, our BN structure presented complex interactions among variables visually, which helped in understanding trigger mechanisms of occurrence of different postvaccination symptoms, although their causality still warrant further verification.

Although all the variables contained in the network structure were used to predict GBS, we also calculated a simplified posttest probability of GBS using only information regarding three GBS parent nodes (age, gender, and erythema) and obtained some interesting results. A highly cited meta-analysis integrated 16 original GBS-related studies and obtained a generalized estimate of incidence; the age-specific estimates showed that GBS incidence increased by 20% for every 10-year increase in age [4]. However, we found that the risk of GBS first increased and then decreased with increasing age, based on VAERS data, peaking in the age group of 50-64 years and declining in the age group of ≥65 years. In fact, several articles reported a similar U-shaped relationship between age and GBS [29-32,36,37], whereas the random-effects negative binomial regression model the researchers used did not detect this fluctuation [4]. In line with previous studies [29-32], our study also found that men had a higher risk of GBS than women. As for the negative correlation we found between erythema and GBS, we searched medical archives extensively and a study pointed out that intermittent erythema in GBS was quite rare and should be recognized as a rare manifestation of GBS [33]. To sum up, our findings suggested that male vaccinees aged 50-64 years and without erythema should be on the alert or be warned by clinicians about an increased risk of GBS, especially when they also experience symptoms of asthenia, hypesthesia, muscular weakness, or paresthesia.

To our knowledge, this BN model is the second attempt to use VAERS data for GBS risk prediction after our previous logistic regression model [18]. This model performed well at predicting GBS both in internal cross-validation and external validation, with AUC reaching 0.866 and 0.829, respectively, which was superior to the performance of the logistic regression model (0.775 and 0.769, respectively). This superiority originated from the different GBS-related AEs we screened through MB and the complex interactions considered in the BN model. In the external validation, although the established BN model had a barely satisfactory performance with a sensitivity of 0.673, it performed well in specificity (0.854), which is an important index because a higher value is an indication of a model with fewer misdiagnoses. The accuracy in the external validation (0.843) also corroborated that the established BN model is worth trying in medical practice. In addition, the minor differences in performance between the top 50 AEs–based networks and top 100 AEs–based networks illustrated that the BN structure learned from the top 50 AEs had already included the most informative GBS-related AEs. As clinical practice prefers a more compact model, albeit with slightly less predictive power, we primarily reported the BN model learned from the top 50 AEs.

The established BN model not only provides a promising tool for clinicians to assist in decision-making, but it can also be incorporated into a web platform, making it convenient for people who want to monitor their own risk of GBS based on mild symptoms. Furthermore, few input symptoms are needed by the BN model, making it more easily acceptable to the general population, which may facilitate this monitoring behavior. Given the natural progression of GBS, it may evolve to respiratory arrest and death, but the prognosis improves considerably with accurate diagnosis and prompt treatment.

However, there are several limitations to consider. First, both VAERS and the EudraVigilance system are spontaneous reporting systems and accept reports submitted without validation; therefore, reporting biases are inevitable. For example, Medical Dictionary for Regulatory Activities preferred terms annotated by domain experts in VAERS may overlap and reporting may be stimulated by possible publicity. Second, because many AEs are sparse in the annual data, we chose to use data across all years in constructing the BN model. This approach neglected the influence of different formulations of influenza vaccines in different years possibly related to GBS risk. Third, the cohort we analyzed was restricted to the VAERS FLU3 and EudraVigilance influenza vaccines; thus, the BN model should be interpreted and applied with caution and this novel risk prediction model needs to be further studied, validated, and evaluated by prospective studies. Fourth, there may be potential overfitting problems driven by the MLE method; nonetheless, the good performance during the external validation indicated that overfitting issues were controlled well in this study. Finally, BN modelling requires the assumption of the Markov property; thus, some dependence relationships may not be revealed yet in the ultimate network. An interesting future direction is to *quantify* the marginal dependency between the occurrence of GBS and each of the 10 identified AEs that are deemed to be predictive of GBS, using bivariate generalized linear mixed effects models or the transformation-free Sarmanov family [38,39].

### Conclusions

In conclusion, this study developed and externally validated a BN model for GBS risk prediction based on large-scale US and European influenza vaccine postmarketing cohort data. The findings illustrated that a BN model can effectively identify the most significant factors of GBS, improve understanding of the complex interactions among different postvaccination symptoms through its graphical representation, and accurately predict the risk of GBS both in internal and external validation. The established BN model could further assist clinical decision-making by providing an estimated risk of GBS for a specific vaccinee or be developed into an open-access platform for vaccinees’ self-monitoring.

## Appendix

Multimedia Appendix 1 Transparent Reporting of a Multivariable Prediction Model for Individual Prognosis or Diagnosis (TRIPOD) checklist.

Multimedia Appendix 2 The strength of the conditional-dependence relationships between nodes.

## Abbreviations

AE: adverse event
AUC: area under the receiver operating characteristic curve
BN: Bayesian network
CPT: conditional probability table
FLU3: trivalent influenza vaccine
GBS: Guillain-Barré syndrome
MB: Markov blanket
MLE: maximum likelihood estimation
VAERS: Vaccine Adverse Event Reporting System

## References

[1] (2019). Influenza (flu) vaccine (inactivated or recombinant): what you need to know. Centers for Disease Control and Prevention.

[2] Willison HJ, Jacobs BC, van Doorn PA (2016). Guillain-Barré syndrome. Lancet.

[3] Lehmann HC, Hartung HP, Kieseier BC, Hughes RA (2010). Guillain-Barré syndrome after exposure to influenza virus. Lancet Infect Dis.

[4] Sejvar JJ, Baughman AL, Wise M, Morgan OW (2011). Population incidence of Guillain-Barré syndrome: a systematic review and meta-analysis. Neuroepidemiology.

[5] Schonberger LB, Bregman DJ, Sullivan-Bolyai JZ, Keenlyside RA, Ziegler DW, Retailliau HF, Eddins DL, Bryan JA (1979). Guillain-Barre syndrome following vaccination in the National Influenza Immunization Program, United States, 1976--1977. Am J Epidemiol.

[6] Salmon DA, Proschan M, Forshee R, Gargiullo P, Bleser W, Burwen DR, Cunningham F, Garman P, Greene SK, Lee GM, Vellozzi C, Yih WK, Gellin B, Lurie N, H1N1 GBS Meta-Analysis Working Group (2013). Association between Guillain-Barré syndrome and influenza A (H1N1) 2009 monovalent inactivated vaccines in the USA: a meta-analysis. Lancet.

[7] Sivadon-Tardy V, Orlikowski D, Porcher R, Sharshar T, Durand MC, Enouf V, Rozenberg F, Caudie C, Annane D, van der Werf S, Lebon P, Raphaël JC, Gaillard JL, Gault E (2009). Guillain-Barré syndrome and influenza virus infection. Clin Infect Dis.

[8] Sejvar JJ, Pfeifer D, Schonberger LB (2011). Guillain-barré syndrome following influenza vaccination: causal or coincidental?. Curr Infect Dis Rep.

[9] Hughes RA, Wijdicks EF, Benson E, Cornblath DR, Hahn AF, Meythaler JM, Sladky JT, Barohn RJ, Stevens JC, Multidisciplinary Consensus Group (2005). Supportive care for patients with Guillain-Barré syndrome. Arch Neurol.

[10] Centres for Disease Control and Prevention, Food and Drug Administration Vaccine adverse event reporting system.

[11] Lasky T, Terracciano GJ, Magder L, Koski CL, Ballesteros M, Nash D, Clark S, Haber P, Stolley PD, Schonberger LB, Chen RT (1998). The Guillain-Barré syndrome and the 1992-1993 and 1993-1994 influenza vaccines. N Engl J Med.

[12] Haber P, DeStefano F, Angulo FJ, Iskander J, Shadomy SV, Weintraub E, Chen RT (2004). Guillain-Barré syndrome following influenza vaccination. JAMA.

[13] Esposito S, Longo MR (2017). Guillain-Barré syndrome. Autoimmun Rev.

[14] van Koningsveld R, Steyerberg EW, Hughes RA, Swan AV, van Doorn PA, Jacobs BC (2007). A clinical prognostic scoring system for Guillain-Barré syndrome. Lancet Neurol.

[15] Walgaard C, Lingsma HF, Ruts L, Drenthen J, van Koningsveld R, Garssen MJ, van Doorn PA, Steyerberg EW, Jacobs BC (2010). Prediction of respiratory insufficiency in Guillain-Barré syndrome. Ann Neurol.

[16] Walgaard C, Lingsma HF, Ruts L, van Doorn PA, Steyerberg EW, Jacobs BC (2011). Early recognition of poor prognosis in Guillain-Barre syndrome. Neurology.

[17] Rajabally YA, Uncini A (2012). Outcome and its predictors in Guillain-Barre syndrome. J Neurol Neurosurg Psychiatry.

[18] Luo C, Jiang Y, Du J, Tong J, Huang J, Lo Re 3rd V, Ellenberg SS, Poland GA, Tao C, Chen Y (2021). Prediction of post-vaccination Guillain-Barré syndrome using data from a passive surveillance system. Pharmacoepidemiol Drug Saf.

[19] Ghahramani Z (2015). Probabilistic machine learning and artificial intelligence. Nature.

[20] Lee S, Ybarra N, Jeyaseelan K, Faria S, Kopek N, Brisebois P, Bradley JD, Robinson C, Seuntjens J, El Naqa I (2015). Bayesian network ensemble as a multivariate strategy to predict radiation pneumonitis risk. Med Phys.

[21] Li M, Liu Z, Li X, Liu Y (2019). Dynamic risk assessment in healthcare based on Bayesian approach. Reliab Eng Syst Saf.

[22] Sohn S, Larson DW, Habermann EB, Naessens JM, Alabbad JY, Liu H (2017). Detection of clinically important colorectal surgical site infection using Bayesian network. J Surg Res.

[23] Shen Y, Zhang L, Zhang J, Yang M, Tang B, Li Y, Lei K (2018). CBN: constructing a clinical Bayesian network based on data from the electronic medical record. J Biomed Inform.

[24] Park E, Chang HJ, Nam HS (2018). A Bayesian network model for predicting post-stroke outcomes with available risk factors. Front Neurol.

[25] Collins GS, Reitsma JB, Altman DG, Moons KG (2015). Transparent reporting of a multivariable prediction model for individual prognosis or diagnosis (TRIPOD): the TRIPOD statement. BMJ.

[26] Vaccine Adverse Event Reporting System. U.S. Department of Health & Human Services.

[27] Korb KB, Nicholson AE, Korb KB, Nicholson AE (2010). Introducing Bayesian networks. Bayesian artificial intelligence. 2nd Edition.

[28] Glover F (1990). Tabu search: a tutorial. Interfaces.

[29] Sedano MJ, Calleja J, Canga E, Berciano J (1994). Guillain-Barré syndrome in Cantabria, Spain. An epidemiological and clinical study. Acta Neurol Scand.

[30] Cheng Q, Jiang GX, Fredrikson S, Link H, De Pedro-Cuesta J (2000). Incidence of Guillain-Barré syndrome in Sweden 1996. Eur J Neurol.

[31] Chiò A, Cocito D, Leone M, Giordana MT, Mora G, Mutani R, Piemonte and Valle d'Aosta Register for Guillain-Barré Syndrome (2003). Guillain-Barré syndrome: a prospective, population-based incidence and outcome survey. Neurology.

[32] Cuadrado JI, de Pedro-Cuesta J, Ara JR, Cemillán CA, Díaz M, Duarte J, Fernández MD, Fernandez O, García-López F, García-Merino A, Velasquez JM, Martínez-Matos JA, Palomo F, Pardo J, Tobías A, Spanish GBS Epidemiological Study Group (2004). Public health surveillance and incidence of adulthood Guillain-Barré syndrome in Spain, 1998-1999: the view from a sentinel network of neurologists. Neurol Sci.

[33] Tomari K, Suzuki H, Miyama S (2018). Intermittent erythema in Guillain-Barré syndrome. Pediatr Neurol.

[34] de Andrade da Silva R, Cremaschi RC, Rebello Pinho JR, de Oliveira JB, Coelho FM (2019). Guillain-Barré syndrome-the challenge of unrecognized triggers. Neurol Sci.

[35] Martins H, Mendonça J, Paiva D, Fernandes C, Cotter J (2020). An overlapping case of Miller Fisher syndrome and the pharyngeal-cervical-brachial variant of Guillain-Barré syndrome. Eur J Case Rep Intern Med.

[36] Clinical E, Neurology EP (1998). Guillain-Barré syndrome variants in Emilia-Romagna, Italy, 1992-3: incidence, clinical features, and prognosis. Emilia-Romagna study group on Clinical and Epidemiological Problems in Neurology. J Neurol Neurosurg Psychiatry.

[37] Cuadrado JI, de Pedro-Cuesta J, Ara JR, Cemillán CA, Díaz M, Duarte J, Fernández MD, Fernández O, García-López F, García-Merino A, García-Montero R, Martínez-Matos JA, Palomo F, Pardo J, Tobías A, Spanish GBS Epidemiological Study Group (2001). Guillain-Barré syndrome in Spain, 1985-1997: epidemiological and public health views. Eur Neurol.

[38] Chen Y, Chu H, Luo S, Nie L, Chen S (2015). Bayesian analysis on meta-analysis of case-control studies accounting for within-study correlation. Stat Methods Med Res.

[39] Sarmanov OV (1966). Generalized normal correlation and two-dimensional Frechet classes. Dokl Akad Nauk SSSR.

