# Supplementary material for: A Bayesian Network to Predict the Risk of Post Influenza Vaccination Guillain-Barré Syndrome: Development and Validation Study
Source: JMIR Public Health Surveill. 2022 Mar 25;8(3):e25658. doi: 10.2196/25658 (PMC8994148; doi:10.2196/25658)
Supplement: Multimedia Appendix 2 [file publichealth_v8i3e25658_app2.docx]

**Table S1. The strength of the conditional-dependence relationships between nodes.**

| from | to | strength |
| --- | --- | --- |
| Paresthesia | Hypesthesia | -1174.19 |
| GBS | Paresthesia | -473.905 |
| Age | Paresthesia | -459.514 |
| GBS | Asthenia | -351.293 |
| Dizziness | Asthenia | -258.849 |
| GBS | Muscular weakness | -224.854 |
| GBS | Hypesthesia | -216.104 |
| Hypesthesia | Muscular weakness | -130.524 |
| Chills | Asthenia | -123.137 |
| Nausea | Asthenia | -97.6114 |
| Myalgia | Asthenia | -91.9230 |
| Gender | GBS | -84.7227 |
| Age | Hypesthesia | -73.4747 |
| Pain in extremity | Muscular weakness | -61.0023 |
| Age | GBS | -55.1487 |
| Pain in extremity | Hypesthesia | -32.8805 |
| Erythema | GBS | -30.6230 |
| Paresthesia | Muscular weakness | -28.3502 |

*Note.* The strength of the conditional-dependence relationships was measured using Bayesian information criterion score gain or loss, which would be caused by each arc’s removal. Therefore, negative values correspond to decreases in the network score and positive values correspond to increases in the network score (i.e. the stronger the relationship, the more negative the difference).
